# A Jasmonate-Inducible Defense Trait Transferred from Wild into Cultivated Tomato Establishes Increased Whitefly Resistance and Reduced Viral Disease Incidence

**DOI:** 10.3389/fpls.2016.01732

**Published:** 2016-11-22

**Authors:** Rocío Escobar-Bravo, Juan M. Alba, Clara Pons, Antonio Granell, Merijn R. Kant, Enrique Moriones, Rafael Fernández-Muñoz

**Affiliations:** ^1^Instituto de Hortofruticultura Subtropical y Mediterránea “La Mayora”, Universidad de Málaga – Consejo Superior de Investigaciones CientíficasAlgarrobo-Costa, Spain; ^2^Department of Population Biology, Institute for Biodiversity and Ecosystem Dynamics, University of AmsterdamAmsterdam, Netherlands; ^3^Instituto de Biología Molecular y Celular de Plantas, Consejo Superior de Investigaciones Científicas – Universidad Politécnica de ValenciaValencia, Spain

**Keywords:** acylsugars, *Bemisia tabaci*, plant breeding, methyl jasmonate, tomato, *Tomato yellow leaf curl virus*, trichomes, whitefly

## Abstract

Whiteflies damage tomatoes mostly via the viruses they transmit. Cultivated tomatoes lack many of the resistances of their wild relatives. In order to increase protection to its major pest, the whitefly *Bemisia tabaci* and its transmitted *Tomato Yellow Leaf Curl Virus* (TYLCV), we introgressed a trichome-based resistance trait from the wild tomato *Solanum pimpinellifolium* into cultivated tomato, *Solanum lycopersicum*. The tomato backcross line BC_5_S_2_ contains acylsucrose-producing type-IV trichomes, unlike cultivated tomatoes, and exhibits increased, yet limited protection to whiteflies at early development stages. Treatment of young plants with methyl jasmonate (MeJA) resulted in a 60% increase in type-IV trichome density, acylsucrose production, and enhanced resistance to whiteflies, leading to 50% decrease in the virus disease incidence compared to cultivated tomato. Using transcriptomics, metabolite analysis, and insect bioassays we established the basis of this inducible resistance. We found that MeJA activated the expression of the genes involved in the biosynthesis of the defensive acylsugars in young BC_5_S_2_ plants leading to enhanced chemical defenses in their acquired type-IV trichomes. Our results show that not only constitutive but also these inducible defenses can be transferred from wild into cultivated crops to aid sustainable protection, suggesting that conventional breeding strategies provide a feasible alternative to increase pest resistance in tomato.

## Introduction

Tomato (*Solanum lycopersicum* L.) is one of the most important and consumed vegetables worldwide. Production, however, is severely threatened since tomatoes are susceptible to diseases caused by all type of pathogens (i.e., viruses, bacteria, and fungi), and animal pests such as nematodes, insects, and mites. The low level of genetic variation within the cultivated species makes difficult to find natural sources of resistance for breeding programs. This is due to the consecutive strong genetic bottlenecks performed during domestication, where marketable genotypes were selected by using low numbers of plants from the existing germplasms (Bai and Lindhout, 2007). Consequently, current cultivated tomato varieties present a large diversity in growth habit, leaf morphology and fruits-related traits, but they lack resistances to pests and diseases (Schauer et al., 2005; Foolad, 2007). One of the major arthropod pests of cultivated tomato is the whitefly (Hemiptera: Aleyrodidae) *Bemisia tabaci* [Gennadius]. Whitefly feeding affects plant development (Inbar and Gerling, 2008) but, most importantly, results in acquisition and transmission of several viral plant diseases (Navas-Castillo et al., 2011). Whitefly-transmitted virus diseases have become an emerging problem in vegetable production for which no efficient solutions have been developed (Moriones and Navas-Castillo, 2000; Navas-Castillo et al., 2011). One of the most destructive of these viral diseases in tomato is Tomato yellow curl disease (TYLCD) caused by *Tomato yellow leaf curl virus* (TYLCV) (Moriones and Navas-Castillo, 2009). Host–plant resistance may be decisive to conduct successful integrated pest management (IPM) of whiteflies in order to prevent plant viral diseases (Nombela and Muñiz, 2009). This notion is reinforced by the observation that insect-transmitted virus diseases are more restricted when the performance of their vectors is lower (Mutschler and Wintermantel, 2006; Broekgaarden et al., 2011). In this sense, plant breeding for insect resistance has received an increased interest since resistances from wild tomato species can be introgressed into susceptible cultivars (Foolad, 2007).

Resistance to herbivores largely depends on physical and chemical plant defenses that deter or inhibit feeding, oviposition and development of larvae or adults. Among these resistance traits, presence of glandular trichomes and their production of allelochemicals – e.g., acylsugars, sesquiterpenes, and methyl ketones – found in wild tomato species have been shown to confer a high level of resistance against whiteflies (Mutschler et al., 1996; Freitas et al., 2002; Muigai et al., 2002; Bleeker et al., 2009; Firdaus et al., 2012). Cultivated tomato lacks many of these secondary metabolites and its glandular trichomes produce insufficient levels of anti-herbivore substances making them relatively susceptible to a wide range of pests (Besser et al., 2009; McDowell et al., 2011). Only a few breeding programs for plant resistance to whiteflies were successful in transferring constitutive trichome-based resistances from wild into susceptible cultivated tomatoes (Goffreda et al., 1989; Maluf et al., 2010; Leckie et al., 2012), but to our knowledge no such commercial cultivar has been released commercially yet.

Previously we reported that the wild tomato species *Solanum pimpinellifolium* accession TO-937 was resistant to the two-spotted spider mite *Tetranychus urticae* and the whitefly *B. tabaci* (Fernández-Muñoz et al., 2000; Alba, 2006; Rodríguez-López et al., 2011). Genetic and biochemical studies revealed that resistance in TO-937 was associated with acylsucroses-producing type-IV glandular leaf trichomes (Fernández-Muñoz et al., 2003; Alba et al., 2009). The close phylogenetic relationship of this wild species with the cultivated tomato made TO-937 suitable as a resistance donor. Accordingly, acylsucrose-producing advanced backcross (BC) lines were generated (at BC_3_ and BC_5_ levels of introgression) using the tomato (*S. lycopersicum*) cultivar ‘Moneymaker’ as a recipient for the trait. The line in BC_3_ (ABL 14-8) was tested for resistance to whitefly and TYLCV transmission (Rodríguez-López et al., 2011). Results showed that this acylsucrose-producer line was repellent to whiteflies and this reduced the incidence of TYLCD. However, in ABL 14-8, as well as in more advanced backcross lines, the density of acylsucroses-producing type-IV trichomes varied across different plant ages. Therefore, low levels of acylsucrose production were detected in young plants. This resulted in a limited protection against whiteflies and lower effectiveness in reducing TYLCD transmission in 4–5 leaf stage ABL 14-8 plants (Rodríguez-López et al., 2011). Because, TYLCD infections during early growth stages may have a devastating effect on tomato yield (Moriones and Navas-Castillo, 2000; Srinivasan et al., 2012), it was necessary to obtain plants displaying an earlier expression of these resistance traits. We reasoned that an alternative approach, i.e., by artificially inducing such trichomes in early growth stages of BC lines, might enhance plant protection. Artificial induction of plant defenses against herbivores can be accomplished by the application of elicitors, such as plant hormones (Thaler et al., 1999; Andreu et al., 2006). For example, expression of anti-herbivore direct and indirect defenses such as the biosynthesis of defensive secondary metabolites and enzymes (Takabayashi et al., 2000; Kant et al., 2004; Chen et al., 2006), and trichome production (Boughton et al., 2005; Campos et al., 2009; Maes and Goossens, 2010) can be induced by the plant hormone jasmonic acid (JA) or its derivative volatile form methyl jasmonate (MeJA).

In the present study, we investigated whether MeJA application might promote an early expression of the TO-937 derived trichome-based resistance traits against whiteflies present in an acylsucrose-producing BC_5_ line, BC_5_S_2_. Using the two near-isogenic lines, i.e., BC_5_S_2_ and its recurrent parent ‘Moneymaker,’ we assessed to which extent MeJA induces the trichome-based defense traits of TO-937 and if such induction suffices for enhancing protection against whiteflies and the viral disease they transmit, TYLCD. For this we tested the inducibility of type-IV trichome densities and exudates, and the effect on tomato resistance against whiteflies. Moreover, we assessed the transcriptomic profiles of BC_5_S_2_ and ‘Moneymaker’ in order to provide a mechanistic explanation for the differences observed between the two plant lines. Here, we show that the constitutive defenses based on acylsucrose-producing type-IV trichomes can be modulated by MeJA in the tomato breeding line BC_5_S_2_, but not in ‘Moneymaker.’ We argue that we have transferred an MeJA-inducible defense from a wild tomato into a cultivated breeding line.

## Materials and Methods

### Plant Material and Hormone Treatments

The tomato cultivar ‘Moneymaker’ (MM) and its near-isogenic line BC_5_S_2_ were used for the experiments. BC_5_S_2_ was generated from the initial cross *S. lycopersicum* cv. Moneymaker ×*S. pimpinellifolium* acc. TO-937 followed by five cycles of combined recurrent crosses toward ‘Moneymaker’ and subsequent selfing steps with selection for high type-IV trichome density and acylsugar production, plus two additional final selfing steps (Supplementary Figure S1). Seedlings were sown in plastic pots of 12 cm containing 15% plant-nutrient loaded zeolite and 85% coconut fiber substrate. Plants were grown within a glasshouse under natural lighting with loose temperature control (22–27°C day, 17–20°C night) and watered when needed. Two leaf growth-stage plantlets were sprayed with 7.5 mM methyl jasmonate (MeJA) (Sigma-Aldrich) in 0.8% ethanol aqueous solution until the point of run-off. Mock treatment with 0.8% ethanol aqueous solution was used as control. Multiple MeJA inductions were performed by three applications on days 0, 7, and 14.

### Whitefly Colony and Virus Inoculation

Non-viruliferous *B. tabaci* whiteflies [Mediterranean (MED) species, formerly Q biotype] originating from field individuals collected in Málaga (Spain) were reared on melon plants (*Cucumis melo* L. ‘ANC42,’ IHSM La Mayora-CSIC germplasm collection; or cultivar Primal F_1_, S&G Vegetables) within wooden cages covered with insect-proof nets, in a glasshouse with loose temperature control (22–27°C day, 17–20°C night) and supplemental light when needed. Viruliferous whiteflies were obtained by allowing *B. tabaci* adults a 48-h acquisition access period (AAP) on TYLCV-infected MM plants. The infectious clone of the worldwide distributed IL strain of TYLCV begomovirus associated to TYLCD (TYLCV-IL) (Morilla et al., 2005) was used to infect tomato plants by using the *Agrobacterium tumefaciens*-mediated inoculation (further on referred to as “agroinoculation”) method described in Monci et al. (2005). Detection of TYLCV-IL was performed by squash-blot hybridization following the indications described by Navas-Castillo et al. (2000).

### MeJA Effect on Glandular Trichome Induction and Targeted Trichome-Associated Secretions

Trichome density and targeted associated secretions in leaf exudates were measured on leaflets of the third youngest leaf at 21 days after the initial hormone treatment (dai). Two independent replicated experiments were carried out. Type-IV trichome density was measured following the indications by Alba et al. (2009). Previous analysis of TO-937 and the derived *S. lycopersicum* introgression lines indicated that these produced sucrosyl esters. Epicuticular leaf acylsucroses were extracted and de-esterified using the method described by Goffreda et al. (1990), and the resulting free-sugar moiety was quantified spectrophotometrically using a hexokinase-based glucose assay. In short, aliquots of acylsucroses were concentrated by evaporation, re-dissolved in methanol and saponified adding 0.04 N NaOH. Free sucrose was hydrolyzed to glucose and fructose by adding invertase (Sigma-Aldrich), and then phosphorylated by adenosine triphosphate (Sigma-Aldrich) in the reaction catalyzed by hexokinase (Sigma-Aldrich). The resulting glucose-6-phosphate was oxidized to 6-phosphogluconate in the presence of nicotinamide adenine dinucleotide in a reaction catalyzed by glucose-6-phosphate dehydrogenase (Sigma-Aldrich). The increase in absorbance at 340 nm was recorded, and initial sucrose quantities were determined by using a sucrose standard curve in the range of 0.15–5.8 mM, and expressed as nmol of sucrose esters per cm^2^ of leaf area. Phenolic compounds from leaf exudates were extracted by using a method described by Antonious et al. (2003) and quantified using the Folin-Ciocalteu colorimetric method (Hagerman et al., 1997) (for more details see Supplementary Method 1). The concentration of phenolics was determined using chlorogenic acid as a standard, and expressed as chlorogenic acid equivalents in micrograms per cm^2^ of leaf area. In addition, phenolics were visualized locally *in planta* by diphenylboric acid-2-aminoethyl ester (DPBA) staining following the indications described by Buer et al. (2007).

### Whitefly Preference Bioassays

Whitefly settling behavior was assessed under no-choice conditions following the experimental design described by Rodríguez-López et al. (2012). Six detached leaflets of control and MeJA-treated MM or BC_5_S_2_ plants were collected at 21 dai and placed inside a plastic tray (25 cm × 25 cm) forming a circle. The petiolules of the leaflets were inserted in perforated small petri dishes filled with nutrient solution (0.25 g/l of Nutrichem 60, Miller Chemical, Hanover, PA, USA). Thirty adult *B. tabaci* whiteflies were then released in the center of the circle after a short cold treatment. Plastic trays were closed with a lid where a perforated hole covered with muslin allowed ventilation. The trays were placed in a growth chamber (25°C and 16:8°h photoperiod). Number of whiteflies settled on each leaflet side was recorded at 0.5, 1, 2, 4, 8, 24, and 48 h after release. Six replicates (i.e., six trays per genotype and treatment) of this experiment were performed.

### TYLCV Primary Transmission Experiments

An experiment to assess TYLCD primary transmission was conducted following the no-choice design described by Rodríguez-López et al. (2011). For this, 21 days after the initial mock and MeJA treatments, MM and BC_5_S_2_ plants were placed within separated walk-in insect-proof net structures (5 m length × 5 m width × 2 m height). Then, twenty TYLCV-IL-viruliferous *B. tabaci* individuals per plant were released at the center of a circle (2 m of diameter) of 22 control or MeJA-treated MM or BC_5_S_2_ plants. After 48 h, insects were eliminated by insecticide treatment, and plants were moved to a glasshouse until analysis. Detection of TYLCV-IL infection was performed in the youngest newly emerged leaf at 7, 14, 21, and 28 days post-inoculation (dpi).

### Microarray Analysis

RNA was isolated from young leaves of multiple MeJA-treated and mock-treated MM and BC_5_S_2_ plants at 21 dai (hormone treatments applied at days 0, 7, and 14) by using RNeasy Mini kit (Qiagen) followed by DNase treatment (Ambion) and checked for integrity and quality. Three biological replicates of each treatment were fragmented, labeled and hybridized to the EUTOM3 tomato exon array according to the manufacturer’s instructions (Affymetrix^®^, Santa Clara, CA, USA) at Unitat Central d’Investigació (Universitat de Valencia, Spain), as described in Powell et al. (2012). Microarray design and experimental data are available in the ArrayExpress database^1^ under the accession numbers of A-MEXP-2227 and E-MTAB-2898, respectively.

Up-regulated or down-regulated genes at least 1.5-fold greater in MeJA-treated BC_5_S_2_ than in mock-treated BC_5_S_2_ plants and in MeJA-treated MM than mock-treated MM plants were first selected (FDR-corrected *P*-value ≤ 0.05 for both the ANOVA and the *post hoc* test). Then, a second statistics filter was used to select genes up-regulated or down-regulated more in MeJA-treated BC_5_S_2_ than in MeJA-treated MM plants. For a more detailed description of the design and analysis see Supplementary Method 2. The identified differentially expressed genes classified into clusters were used to calculate MapMan term enrichment scores against molecular function categories by applying Fisher Exact tests using a local, customized version of the ‘catscore.pl’ Perl script described in Cheung et al. (2003). MapMan terms with a *P* < 0.05, and more than three regulated genes for the Mapman-term were defined as over-represented (Supplementary Data sheet 1).

### Gene Expression Analysis by RT-qPCR

To test the early MeJA-mediated induction of acylsugar metabolism-related genes in the BC_5_S_2_ genotype, semi-quantitative RT-PCR analyses were first conducted (data not shown) at 1 day after initial MeJA application. Genes up-regulated according to the microarray analysis, together with other acylsugar biosynthesis-related genes reported as highly expressed in type-IV glandular trichomes of the acylsugar-producing wild tomato species *Solanum pennellii* (Slocombe et al., 2008) were analyzed. Those genes showing higher up-regulation were then analyzed by RT-qPCR in a time-course experiment. For this, BC_5_S_2_ and MM plants were multiple treated with MeJA or mock solution at days 0, 7, and 14. Samples for gene expression analysis were taken at 1, 3, 7, 8, 14, and 15 days after initial induction (dai). Three leaflets per plant were detached and homogenized in liquid nitrogen and total RNA was isolated using a phenol LiCl-based method (Verdonk et al., 2003) and treated with DNase (Ambion). First strand cDNA was synthesized from 4 μg of total RNA using M-MuLV Reverse Transcriptase (Fermentas), as described by the manufacturer, in a 20 μl reaction. PCR was performed in ABI7500 Real-Time PCR system (Applied Biosystems) using Platinum SYBR Green qPCR SuperMix-UDG (Invitrogen). PCR reactions of 20 μl contained 0.25 μM of each primer, 0.1 μl ROX reference dye and 1 μl of cDNA. The cycling program was set to 5 min at 50°C, 2 min at 95°C, 40 cycles of 15 s at 95°C and 1 min at 60°C, followed by a melting curve analysis. Five biological replicates (i.e., each obtained from an individual plant) were analyzed per treatment and time point with two technical qRT-PCR replicates per individual sample. Expression levels for all the genes were normalized to the expression of the endogenous control gene *Actin*. The normalized expression (NE) data were calculated by the ΔCt method NE = -(PE_target_ˆCt_target_)/(PE_reference_ˆCt_reference_) (PE = primer efficiency; Ct = cycle threshold). The PEs were determined by fitting a linear regression on the Ct-values of a standard cDNA dilution series. To plot the relative expression, NE values were scaled the lowest average NE within the plot, being the lowest average in each plot set to 1. The gene-specific primers used for the PCRs are listed in Supplementary Table S1.

### Effect of Whitefly Infestation on JA-Associated Defenses and Acylsucrose Secretions in MeJA-Induced BC_5_S_2_ Plants

To test the effect of whitefly infestation on JA-induced acylsucrose secretions in BC_5_S_2_, we measured the acylsucrose secretions in leaf exudates and assessed the expression of the JA-marker gene *WIPI-II* (*wound inducible proteinase inhibitor-II*) (called *PI-IIf* in Alba et al., 2015) and the acylsugar biosynthesis-related gene *BCKD-E_2_* in control and MeJA-treated plants after infestation with non-viruliferous *B. tabaci*. To obtain this material, young BC_5_S_2_ plants were treated three times with MeJA or mock solution at 0, 7, and 14 days. At 21 days after the initial hormone or mock treatment, 22 plants per treatment were placed in a circle in separated walk-in insect-proof net structures. Two blocks per treatment were set, each block corresponding to one walk-in insect-proof net structure. *B. tabaci* whiteflies (60 individuals per plant) were then released in the center of the circle of mock-treated or MeJA-treated BC_5_S_2_ plants and allowed to develop a new population until the end of the experiment. Plants were sampled for acylsucrose production and gene expression analysis at 7 and 14 days after whitefly release.

### Statistical Analysis

All data were first analyzed using Levene and Kolmogorov–Smirnov tests to determine the heteroscedasticity of variance and normality, respectively. Differences in type-IV trichome density and acylsugar content in leaf exudates of mock-treated and MeJA-treated BC_5_S_2_ plants were tested by *t-*test. To normalize the data and stabilize the variance, acylsucrose measurements were Log (x+1) transformed prior to analysis. Differences in phenolic content among groups, i.e., mock-treated and MeJA-treated MM and BC_5_S_2_ plants, were tested by generalized linear models (GLMs), using Linear distribution and identity link function, followed by Fisher’s least significant difference (LSD) *post hoc* test. Whitefly preference data were analyzed by GLM using Log (x+1) as the link function and Poisson as the probability distribution. Results of three replicated experiments assessing TYLCD primary transmission were homogeneous, as determined by contingency tables and associated chi-squared test (*P* ≤ 0.05), and pooled data were statistically analyzed. For this, differences between number of infected vs. non-infected BC_5_S_2_ and MM plants were analyzed by GLM using Logit as the link function and Binomial as the probability distribution. Pair-wise comparisons among groups at each time point were performed by LSD *post hoc* test. For the TYLCV agroinoculation experiments, differences between infected vs. non-infected BC_5_S_2_ or MM plants were analyzed by *t*-test at each time point. Data of gene expression analysis by RT-qPCR of acylsugar biosynthesis-related genes for mock-treated and MeJA-treated plants were analyzed by one-way nested ANOVA. For this, univariate analysis was used with hormone treatment and technical replicate nested within hormone treatment as fixed factors. Pooled data of acylsucrose production between non-infested and whitefly infested mock-treated or MeJA-treated BC_5_S_2_ plants were analyzed by Student’s *t-*test. Prior analysis, heterogeneity of pooled data was statistically tested using contingency tables and associated chi-squared test (*P* ≤ 0.05). Differences in *WIPI-II* and *BCKD-E_2_* expression levels between non-infested and whitefly-infested plants were analyzed by one-way nested ANOVA. For this, normalized expression data were Log transformed when needed prior analysis. Univariate analysis was used with whitefly infestation and technical replicate nested within whitefly infestation as fixed factors.

## Results

### MeJA Promotes Type-IV Glandular Trichome Density and Secretion of Acylsugars and Phenolics in BC_5_S_2_

**FIGURE 1:**
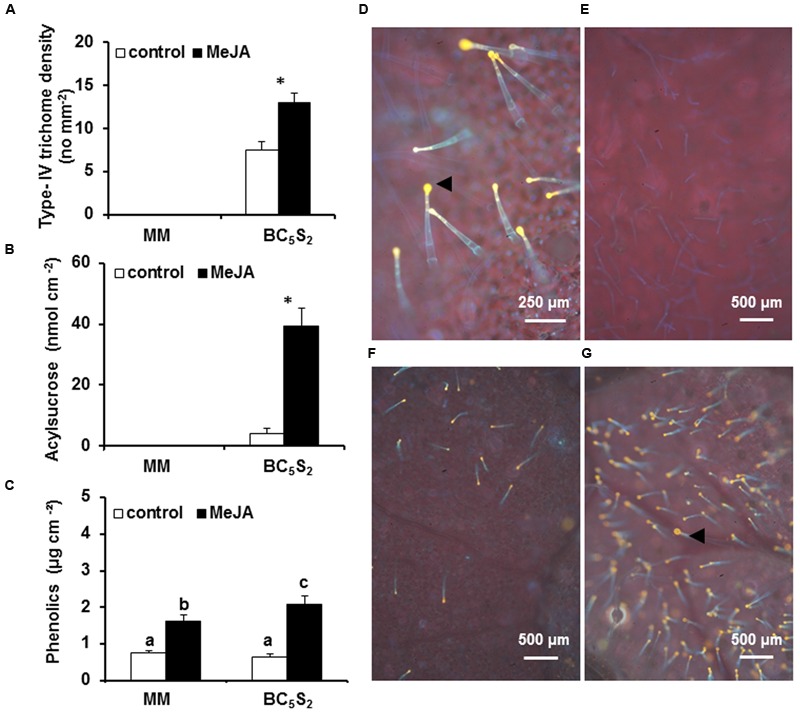
**Effect of multiple methyl jasmonate (MeJA) applications on type-IV glandular trichome density and targeted secretions at 21 days after the initial hormone induction in BC_5_S_2_ and ‘Moneymaker’ (MM).** Two independent experiments were performed. Representative data from one experiment are shown. **(A)** Mean values + SEM (*n* = 12–15) of type-IV glandular trichome densities measured in mock- and MeJA-treated plants. **(B)** Mean values + SEM (*n* = 15) of acylsucrose secretions in mock-treated and MeJA-treated MM and BC_5_S_2_ plants. Significant differences between treatments in BC_5_S_2_ plants were tested by *t-test*. Asterisks denote significant differences at *P ≤* 0.001. **(C)** Mean values + SEM (*n* = 15) of phenolic content in leaf exudates of mock-treated and MeJA-treated MM and BC_5_S_2_ plants. Bars with different letters indicate significant differences among groups compared by Fisher’s least significant differences (LSD) test at *P ≤* 0.05. Representative micrographs of phenolics accumulation in diphenylboric acid-2-aminoethyl ester (DPBA)-flavonoid stained abaxial leaf surfaces of mock-treated **(D,F)** and MeJA-treated **(G)** BC_5_S_2_ plants. Black arrows point golden fluorescence associated to the presence of phenolic compounds in the glands of type-IV trichomes of BC_5_S_2_ plants. **(E)** Absence of type-IV trichomes and gold fluorescence was observed in DPBA-treated abaxial leaf surfaces of MeJA-treated MM plants.

Moneymaker and its near-isogenic line BC_5_S_2_ differ in their leaf trichome composition. In MM, type-I and -VI glandular, but especially type-V non-glandular trichomes, constitute the majority of trichomes on abaxial and adaxial leaf surfaces. Conversely, in BC_5_S_2_ the acylsucrose-producing type-IV glandular trichomes, which are absent in MM, densely cover the abaxial leaf surface; non-glandular type-V, and glandular type-I and type-VI trichomes are also present in BC_5_S_2_ plants albeit at low densities (Supplementary Figure S2). After multiple MeJA applications, type-IV glandular trichome density and acylsucrose production significantly increased in BC_5_S_2_ plants, but MM remained lacking these traits (**Figures 1A,B**). Phenolic compounds were present in the leaf exudates of both genotypes and significantly induced by MeJA (**Figure 1C**). Though, the presence of phenolics was previously associated with type-VI trichomes (Kennedy, 2003), these compounds were also detected in the glands of type-IV trichomes of DPBA-stained BC_5_S_2_ leaves (**Figures 1D,F,G**). No DBPA-fluorescence was detected in abaxial leaf surface of MeJA-treated MM plants (**Figure 1E**).

### Whitefly Preference and TYLCV-IL Primary Transmission Are Reduced in MeJA-Treated BC_5_S_2_ Plants

**FIGURE 2:**
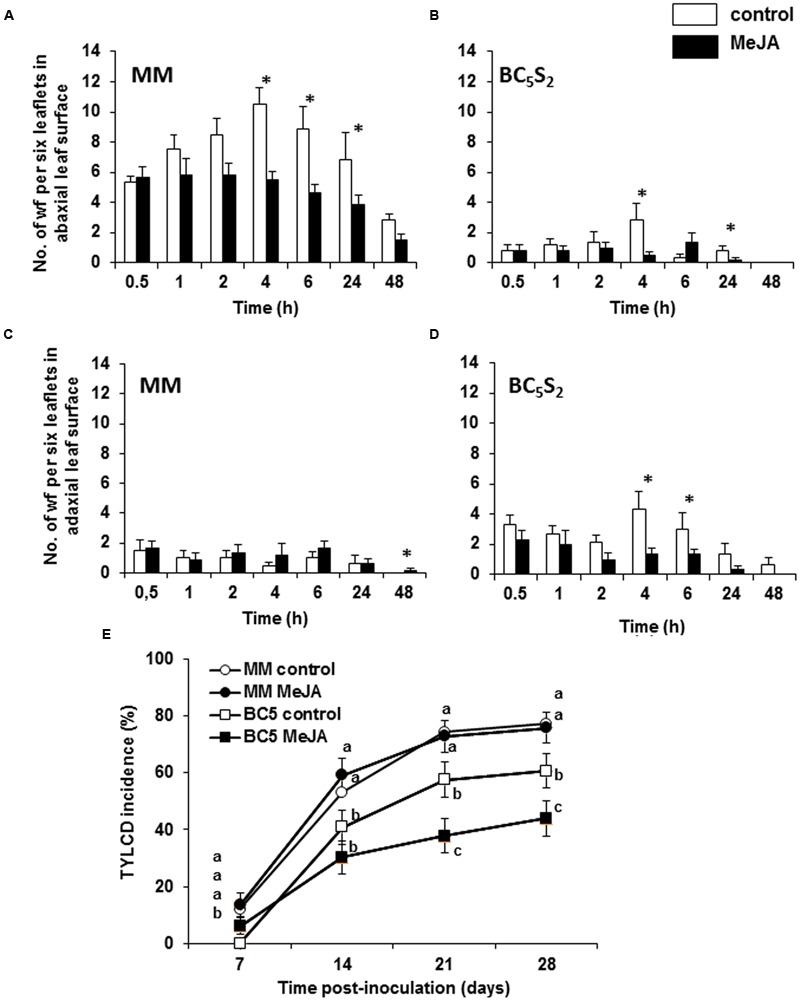
**Evaluation of *Bemisia tabaci* biotype Q preference and Tomato yellow leaf curl disease (TYLCD) primary transmission in mock-treated and multiple methyl jasmonate (MeJA)-treated ‘Moneymaker’ (MM) and BC_5_S_2_ plants under no-choice experimental conditions.** Plants received three MeJA applications at 0, 7, and 14 days. At 21 days after initial hormone treatments whitefly preference and virus primary transmission experiments were performed. Mean number + SEM of whiteflies (wf) per six leaflets settled on adaxial **(A,B)**, and abaxial **(C,D)** leaflet surfaces of mock-treated and MeJA-treated MM and BC_5_S_2_ plants at different time points after release of 30 unsexed adult whiteflies. Asterisks indicate significant differences (*P ≤* 0.05) between treatments at each time point. **(E)** Estimation of primary TYLCD transmission in mock-treated and MeJA-treated BC_5_S_2_ and MM plants. Percentage (mean + SEM) of TYLCV-infected plants at different time points after viruliferous whiteflies were given a 48-h inoculation access period (IAP) are shown. Pooled data of three replicated experiments were homogeneous and are shown in the figure. Pair-wise comparisons among groups at each time point were performed by Fisher’s LSD *post hoc* test. Different letters indicate significant differences among groups at *P* ≤ 0.05.

Lower numbers of whiteflies settled on the leaflets of the acylsugar-producing genotype BC_5_S_2_ when compared to MM (**Figures 2A,B**). When treated with MeJA, however, both MM and BC_5_S_2_ plants were less preferred by whiteflies. In MeJA-treated MM plants, the number of whiteflies settled in abaxial leaf surfaces at 4, 6, and 24 h was significantly lower than in mock-treated controls, though no differences were observed in the adaxial sides (**Figures 2A,C**). Reduced whitefly preference was observed in the abaxial leaf sides of MeJA-treated BC_5_S_2_ plants at 4 and 24 h (**Figure 2B**) and at 4 and 6 h on adaxial leaf sides (**Figure 2D**) when compared to their controls.

**FIGURE 3:**
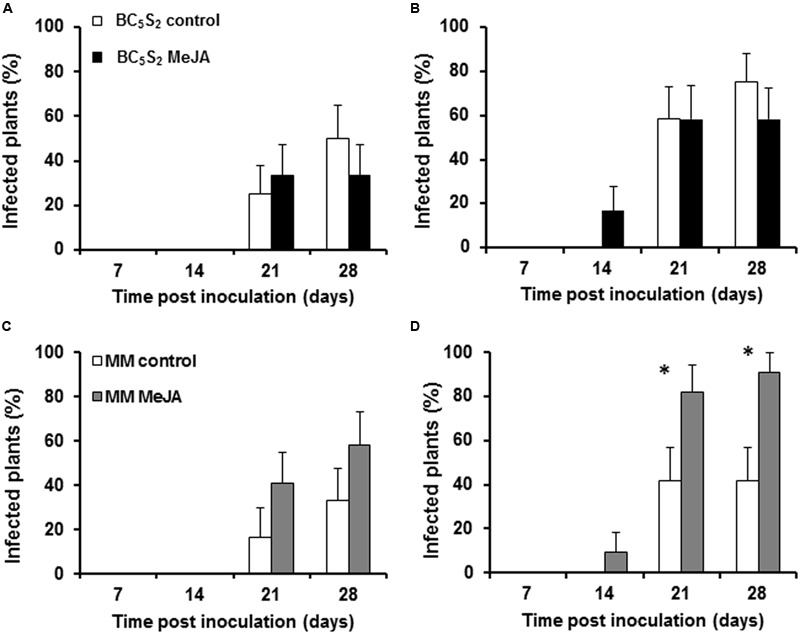
**Effect of Methyl jasmonate (MeJA) applications (on days 0, 7, and 14) on TYLCD incidence in MM and BC_5_S_2_ plants.** TYLCV-IL agroinoculation was performed at 21 days after initial MeJA treatment. Percentage (mean + SEM) (*n* = 12) of infection at several time points after TYLCV-IL agroinoculation of mock-treated and multiple MeJA-treated BC_5_S_2_ **(A,B)** and MM **(C,D)** plants is shown. The experiment was repeated twice, in February **(A–C)** and May **(B,D)**. Number of infected vs. non-infected plants were analyzed by Generalized Linear Models using Logit as the link function and Binomial as the probability distribution. Asterisks indicate significant differences between treatments (*P* < 0.05) for each time point.

To further investigate the impact of the MeJA treatments on the incidence of the whitefly-transmitted TYLCV-IL, primary transmission of the virus was estimated in mock-treated and MeJA-treated MM and BC_5_S_2_ plants at 21 dai (**Figure 2E**). A significant reduction in TYLCV-IL transmission was observed in mock-treated BC_5_S_2_ plants when compared to MM (**Figure 2E**). Conversely, virus transmission was diminished in MeJA-treated BC_5_S_2_ plants when compared to mock-treated BC_5_S_2_ plants at 21 and 28 dpi. In contrast, no such differences were observed between mock-treated and MeJA-treated MM plants at either time point. To determine whether MeJA treatment may affect infectivity by TYLCV-IL, TYLCV-IL agroinoculation of mock-treated and MeJA-treated BC_5_S_2_ and MM plants was conducted at 21 dai (**Figure 3**). No significant reduction in susceptibility to TYLCV-IL was observed in MeJA-treated BC_5_S_2_ plants in the two replicated experiments (**Figures 3A,B**), reinforcing the notion that the reduction of virus transmission observed in MeJA-treated BC_5_S_2_ plants correlates strongly with the enhancement of acylsucrose production and the effect on whiteflies. Higher susceptibility to TYLCV, however, was observed for hormone-treated MM plants in one of the replicated experiments (**Figure 3D**). Activation of JA defenses can disrupt geminivirus infection (Lozano-Durán et al., 2011). As *Agrobacterium tumefaciens* and TYLCV can induce salicylic acid (SA) signaling pathway in plants (Sheikh et al., 2014; Sade et al., 2015; Villarroel et al., 2016), a stronger and negative SA-JA crosstalk might have diminished induced JA defenses and explain the higher susceptibility to TYLCV in the MeJA-induced tomato plants.

### MM and BC_5_S_2_ Show Differences in their Gene Expression Profiles after MeJA Treatments

To get a better understanding of the mechanistic basis of the MeJA-mediated induced resistance to whiteflies, changes in transcriptome profiles of BC_5_S_2_ and its recurrent parent MM was analyzed by means of microarray.

**FIGURE 4:**
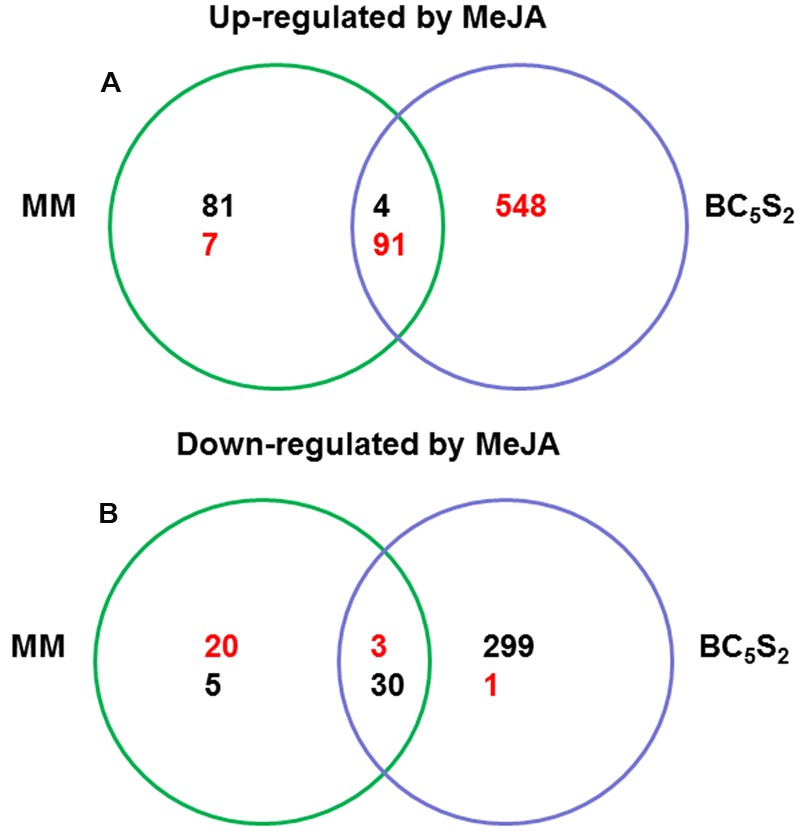
**Venn diagram of genes significantly induced (A)** and repressed **(B)** by multiple methyl jasmonate (MeJA) applications in ‘Moneymaker’ (MM) and BC_5_S_2_ plants at 21 days after the initial hormone treatment. Numbers in red denote genes showing higher expression levels in MeJA-treated BC_5_S_2_ plants when compared to MeJA-treated MM plants. In black, number of genes showing lower expression levels in MeJA-treated BC_5_S_2_ plants when compared to MeJA-treated MM plants.

A total of 2,309 genes (∼26% of EUTOM3 probes) were up- or down-regulated in BC_5_S_2_ and/or MM by MeJA at 21 dai (mean fold change ≥ 1.5 relative to their controls, *P*-value and FDR ≤ 0.05 in both ANOVA test factor and *post hoc* test). Then these genes were filtered and classified according to their ratio between BC_5_S_2_ and MM MeJA- treated plants. Out of them, 1,089 genes were up- or down- regulated by MeJA and showed differences between BC_5_S_2_ and MM MeJA-treated plants higher than 1.5 folds (**Figure 4**). Of these genes, 95 were up-regulated and 33 were down-regulated in both MM and BC_5_S_2_ plants. Functional categories of genes up- or down-regulated by MeJA in both plant genotypes matched to those of JA-, SA-, ethylene- and biotic stress-related genes. In agreement with the observation that MeJA increased the production of surface phenolics in MM and BC_5_S_2_ (**Figure 1C**), aromatic amino acid and phenylpropanoid biosynthesis-related genes were up-regulated in both genotypes. Moreover, our analysis showed the MeJA-induced expression of the AGAMOUS gene *TAG1* in MM and BC_5_S_2_, which has been described to increase glandular trichome density (Pnueli et al., 1994). However, among the genes up-regulated by MeJA in both plant genotypes, 91 showed higher levels of up-regulation in BC_5_S_2_ than in MM plants. This set predominantly (*P* < 0.05 and genes > 3) consisted of genes annotated to be involved in amino acid and lipid metabolism (mainly degradation), secondary metabolism and stress (Supplementary Data sheet 1). This cluster contained several genes related to JA biosynthesis, as well as WRKY transcription factors of which several are involved in the regulation of pathogen/plant immunity, senescence and trichome development (Eulgem et al., 2000; Pandey et al., 2011). In MM and BC_5_S_2_ plants, MeJA treatment down-regulated genes related to protein post-translational modification, including phosphatases and kinases and, among them, genes similar to SOS3- interacting proteins, HAB, ABI1 and PP2C, most of which are negative regulators of ABA signaling in *Arabidopsis* (Santiago et al., 2009). These genes (30 of a total of 33) showed stronger level of down-regulation in BC_5_S_2_ than in MeJA-treated MM plants.

We also found sets of genes differentially expressed in the advanced backcross line BC_5_S_2_ or its recurrent parent MM. Genes that responded to the MeJA-treatment only in BC_5_S_2_ were considered to be the most important candidates directly or indirectly related to the introgressions from TO-937 and, consequently, related to the increase in acylsugar-producing type-IV trichomes observed in response to the hormone treatment (**Table 1**). Indeed, the acylsugar metabolism-related genes *Dihydrolipoyl dehydrogenase, Dihydrolipoyllysine-residue acetyltransferase, 2-isopropylmalate synthase 1* and the *Fatty acid elongase 3-ketoacyl-CoA synthase*, and *Threonine deaminase* were only up-regulated in MeJA-treated BC_5_S_2_ plants. In addition, several tomato trichome-specific acyltransferases, responsible for the acylation of the sucrose backbones of acylsucroses, were up-regulated by MeJA only in BC_5_S_2._ The analysis showed a twofold increase in the expression of the *S. lycopersicum acylsucrose acyltransferase 2* gene (*Sl-ASAT2*) in BC_5_S_2_ after MeJA treatments, and when compared to the expression levels in MeJA-treated MM plants this gene was fourfold more expressed. The product of the *Sl-ASAT2* gene has very recently been described to catalyze, together with another *S. lycopersicum* BAHD [BEAT, AHT, HCBT, DAT (D’Auria, 2006)] acyltransferase, Sl-ASAT1, consecutive reactions to produce di-acylsucrose intermediates (Fan et al., 2016). Interestingly, the BAHD-type acylsucrose acyltransferase gene *Acylsugar acyltransferase3* (*ASAT3*), which was described to catalyze acylation on the five-member furanose ring of diacylsucroses in the tip cells of acylsugar producing type-I trichomes (Schilmiller et al., 2010, 2015) was also induced in MeJA-treated BC_5_S_2_ plants. This gene was twofold induced in BC_5_S_2_ after the hormone treatments, and it was nine times more expressed than in MeJA-treated MM plants. The analysis also showed that BC_5_S_2_ up-regulated more defense -related genes than MM after MeJA treatments, since a higher number of genes involved in biotic and abiotic stress-related responses, signal transduction, and secondary metabolites production were differentially induced only in the former genotype after the hormone treatments. Notably, the effect of MeJA on down-regulation of genes related to the photosynthetic apparatus was also stronger in BC_5_S_2_ when compared to MM.

**Table 1:** Ratio of expression levels (log_2_ transformed; mock-treated *versus* MeJA-treated) of tomato genes involved in acylsugar biosynthesis that are differentially up-regulated by MeJA treatments in the acylsucrose producing breeding line BC_5_S_2_ when compared to the parental line Moneymaker (MM).

Gene name	ID	Ratio MeJA-treated BC_5_S_2_ *versus* MeJA-treated MM	BC_5_S_2_		MM	
			Ratio Mock:MeJA	*P-*value	Ratio Mock:MeJA	*P-*value
*Dihydrolipoyllysine-residue acetyltransferase (BCKD-E_2_)*	Solyc01g066520	1,82	1,681	0,0064^∗∗^	1,156	0,436
*Dihydrolipoyllysine-residue acetyltransferase (BCKD-E_3_)*	Solyc12g099100	2,09	2,369	0,0029^∗∗^	1,288	0,493
*3-ketoacyl-CoA synthase-3 (FAE-3)*	Solyc10g009240	1,84	1,646	0,0056^∗∗^	0,936	0,569
*2-isopropylmalate synthase 1 (IPMS-1)*	Solyc06g053400	1,86	1,626	0,0042^∗∗^	0,945	0,879
*Threonine deaminase (TD)*	Solyc10g083760	1,44	0,968	0,05^∗^	1,437	0,067
*Acylsucrose acyltransferase 2 (SI-ASAT2)*	Solyc04g012020	4,49	1,96	0,0005^∗∗∗^	0,878	0,370
*Acylsugar acyltransferase 3 (SI-ASAT3)*	Solyc11g067270	9,04	2,06	0,028^∗^	0,950	0,999

*Statistically significant differences in expression between mock-treated and MeJA-treated plants are indicated by *P-*values. Asterisks indicate differences at ^∗^*P* ≤ 0.05, ^∗∗^*P <* 0.01 and ^∗∗∗^*P* < 0.001.*

Expression analysis on MeJA-treated MM plants revealed fewer induced or repressed genes in this genotype when compared to those regulated in BC_5_S_2_ after the hormone treatments. A total of 88 genes were up-regulated specifically in MM after MeJA treatment. Out of them, seven genes showed higher expression levels in MeJA-treated BC_5_S_2_ line than in MeJA-treated MM plants, though they were not induced by MeJA in this genotype. Those genes were related to lipid, hormone metabolism, and stress.

To validate the microarray data, four genes whose expression was significantly different between MeJA-treated and control MM and BC_5_S_2_ plants were selected to be re-analyzed via RT-qPCR. The expression levels and differences observed on the microarray matched those observed via RT-qPCR (Supplementary Figure S3; Supplementary Table S2).

### MeJA Induces Expression of Acylsugar Biosynthesis-Related Genes in BC_5_S_2_

**FIGURE 5:**
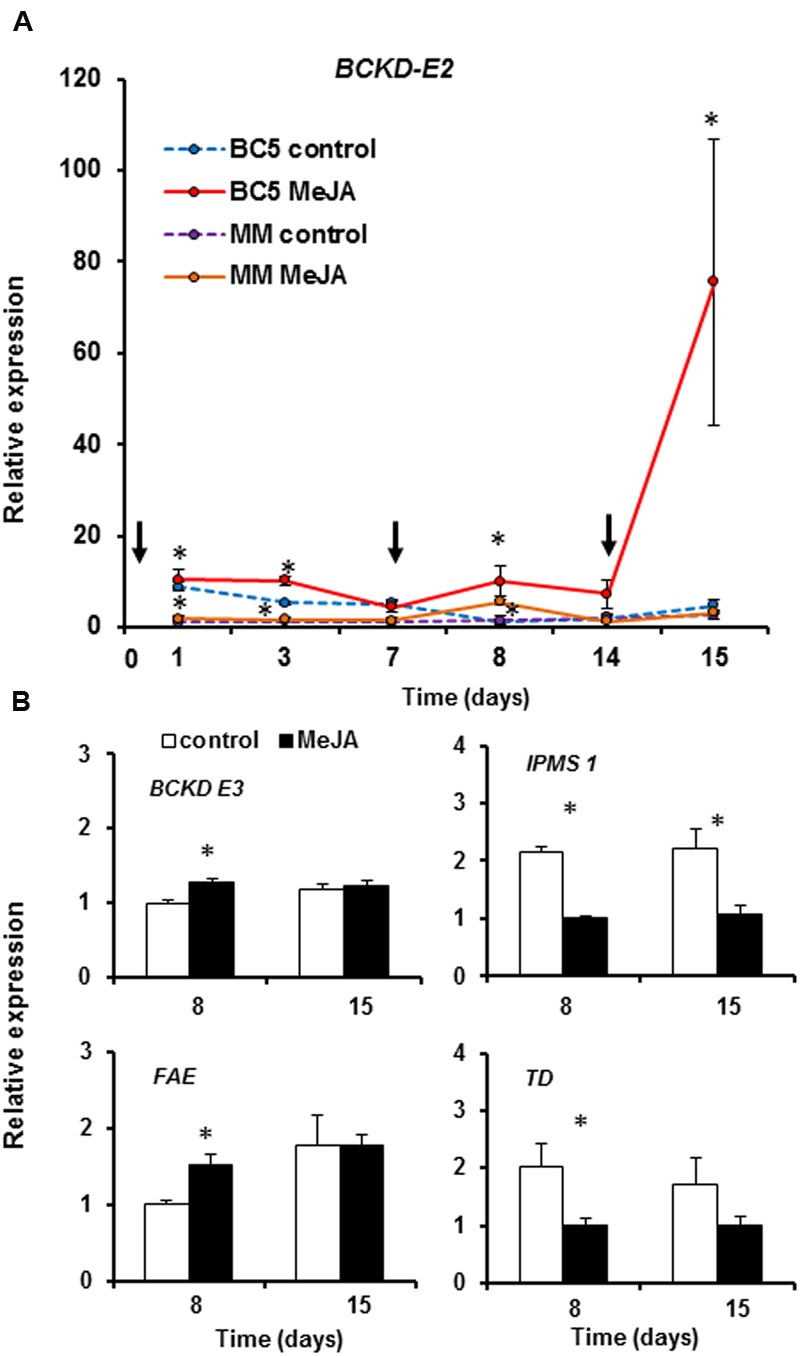
**Relative transcript levels of acylsugar biosynthesis-related genes after multiple methyl jasmonate (MeJA) applications.** Exogenous MeJA was applied at day 0, 7, and 14. **(A)** Time-course expression analysis of the *Dihydrolipoyllysine-residue acetyltransferase* or also called *Branched-chain keto-acid dehydrogenase-E_2_ subunit* (*BCKD-E_2_*) in mock-treated and MeJA-treated BC_5_S_2_ and MM plants at 1, 3, 7, 8, 14, and 15 days after the first MeJA application. Black arrows indicate time-points at which MeJA applications were done. **(B)** Expression analysis performed in BC_5_S_2_ plants at 1 day after the second and third MeJA application (8 and 15 days, respectively) of acylsugar biosynthesis-related genes involved in the following processes: conversion of keto-acids to acyl-CoAs, *Dihydrolipoyllysine-residue acetyltransferase* or also called *Branched chain keto-acid dehydrogenase subunit 3* (*BCKD-E_3_*); fatty acid elongation, *3-ketoacyl-CoA synthase* (*FAE-3*); leucine biosynthesis, *2-isopropylmalate synthase 1* (*IPMS-1*); branched-chain keto-acid precursors biosynthesis, *Threonine deaminase* (*TD*), respectively. The mean + SEM fold induction calculated respect to mock treatment values is shown. Data from five biological replicates and two technical replicates were analyzed and normalized to *Actin* gene. Normalized expression data were Log transformed prior analysis when needed. Differences between mock-treated and MeJA-treated BC_5_S_2_ plants were analyzed separately for each gene and time point by one way nested ANOVA. Asterisks denote significant differences between treatments at *P ≤* 0.05.

The preliminary semiquantitative PCR analysis revealed that *Dihydrolipoyllysine-residue acetyltransferase*, also called *Branched chain keto-acid dehydrogenase subunit 2* (*BCKD-E_2_*) gene (Slocombe et al., 2008), a component of the branched-chain keto-acid dehydrogenase complex (BCKD) responsible for the production of acyl-CoAs, was up-regulated 24 h after the first MeJA application. This gene was then selected for analysis of its expression over time by means of RT-qPCR expression analysis. Expression of *BCKD-E_2_* was induced by MeJA in BC_5_S_2_ plants (**Figure 5A**). A slight induction was detected 1 day after application, which significantly increased at day 3 but ceased again at day 7. After the second MeJA application (day 8), a stronger induction of *BCKD-E_2_* was observed. This induced expression was not maintained over time, with no significant differences observed between mock-treated and MeJA-treated BC_5_S_2_ plants at 14 dai. After the third MeJA application (day 15), the induction of *BCKD-E_2_* was considerably stronger than in previous applications (**Figure 5A**). In MM plants treated with MeJA the *BCKD-E_2_* gene was slightly up-regulated at 1, 3 and 8 dai, albeit at much lower levels than in BC_5_S_2_, and at 15 dai no induction was observed.

We selected the samples from 8 and 15 dai, when expression of *BCKD-E_2_* was higher, to assess the expression levels of four other acylsugar biosynthesis-related genes involved in different steps of this pathway and that according to the microarray analysis were upregulated by MeJA (**Figure 5B**). These four genes were *Dihydrolipoyllysine-residue acetyltransferase* or also called *Branched chain keto-acid dehydrogenase subunit 3* (*BCKD-E_3_*), *3-ketoacyl-CoA synthase* (*FAE-3*), *2-isopropylmalate synthase 1* (*IPMS-1*) and *Threonine deaminase* (*TD*). Our results showed that the *BCKD-E_3_* gene was also significantly up-regulated in MeJA-treated BC_5_S_2_ plants at 8 dai (**Figure 5B**). However, this induction was not as high as for *BCKD-E_2_*, and no significant differences in the expression levels were observed at 15 dai. Similarly, expression of *FAE-3* was significantly up-regulated in MeJA-treated BC_5_S_2_ plants at 8 days, but not at 15 dai. Finally, *IPMS-1* was significantly down-regulated in MeJA-treated BC_5_S_2_ plants at 8 and 15 dai, and *TD* was down-regulated at 8 dai.

### MeJA-Mediated Induction of Defenses in BC_5_S_2_ is Enhanced by Whitefly Infestation

**FIGURE 6:**
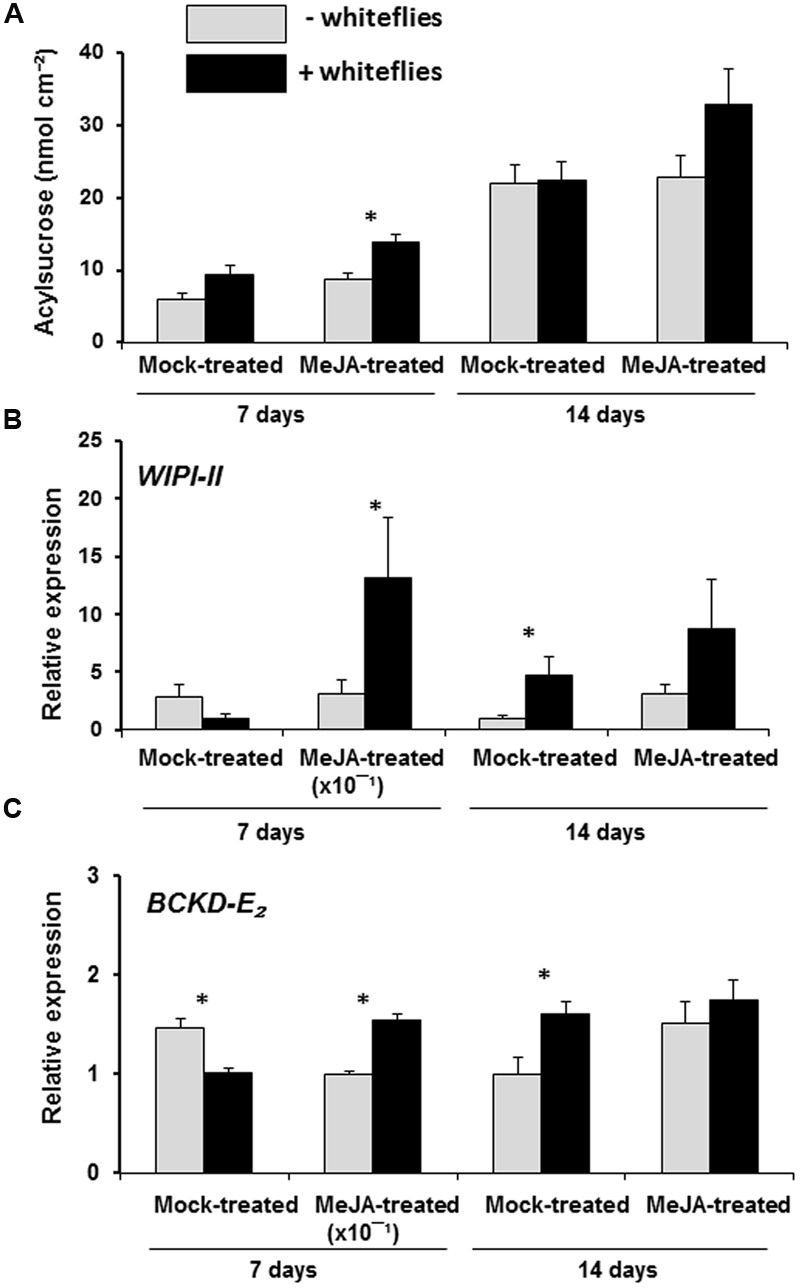
**Effect of *B. tabaci* whitefly infestation on acylsucrose metabolism and JA-mediated signaling defense in mock- and MeJA-treated BC_5_S_2_ plants. (A)** Acylsucrose secretions (mean + SEM, *n* = 21–22) measured in mock-treated and MeJA-treated BC_5_S_2_ plants uninfested (- whiteflies) or infested with *B. tabaci* whiteflies (+ whiteflies) at 7 and 14 days after whitefly release. Pooled data of the two blocks per treament are shown. Asterisks denote significant differences between treatments by *t-*test (*P* ≤ 0.05). **(B)** Gene expression levels of the JA-responsive gene *Wound inducible proteinase inhibitor-II* (*WIPI-II*) and **(C)** the acylsugar metabolism bio-marker gene *Branched-chain keto-acid dehydrogenase-E_2_ subunit*, (*BCKD-E_2_*) were analyzed by real time RT-PCR at 7 and 14 days after whitefly release. Bars indicate mean + SEM fold induction of each treatment group (*n* = 4–5 biological replicates, two technical replicates). Normalized expression data were Log transformed when needed and differences between non-infested and whitefly infested plants were analyzed by one-way nested ANOVA. Univariate analysis was used with whitefly infestation and technical replicate nested within whitefly infestation as fixed factors. Asterisks denote significant differences at *P* ≤ 0.05.

Our results showed that infestation with *B. tabaci* whiteflies induced higher accumulation of acylsucroses in leaf exudates of infested MeJA-treated BC_5_S_2_ plants than in non-infested MeJA-treated plants (**Figure 6A**). Acylsucrose levels, however, did not differ between infested and non-infested mock-treated BC_5_S_2_ plants. To determine whether the enhanced acylsucrose secretion in infested MeJA-treated BC_5_S_2_ plants is accompanied by the induction of JA-mediated responses, expression levels of the JA-marker gene *WIPI-II* and the acylsugar-related gene *BCKD-E*_2_ were assessed at 7 and 14 days after whitefly release. At 7 days, *WIPI-II* was significantly up-regulated in infested MeJA-treated BC_5_S_2_ plants when compared to non-infested MeJA-treated plants (**Figure 6B**). No significant differences were observed at 14 days. However, *WIPI-II* was slightly induced in whitefly-infested mock treated plants at 14 days. Up-regulation of *BCKD-E*_2_ gene coincided with up-regulation of *WIPI-II*. Hence, *BCKD-E_2_* gene was up-regulated in whitefly infested MeJA-treated BC_5_S_2_ plants at 7 days and up-regulated in whitefly-infested mock treated plants at 14 days (**Figure 6C**). At 7 days, however, *BCKD-E_2_* was down-regulated in infested mock-treated BC_5_S_2_ plants which coincided with lower, though not significant, levels of *WIPI-II*.

## Discussion

Here, we have shown that an inducible defense can be transferred from a wild tomato into a commercial variety such that it increases protection against a virus-transmitting whitefly in young plants of the generated BC_5_S_2_ breeding line. In particular, we transferred MeJA-inducible type-IV trichomes and acylsugar production associated to these trichomes into the cultivar ‘Moneymaker’ thus rendering the plants less attractive to whiteflies and deceasing TYLCV disease incidence.

First, our results show that repeated MeJA applications on MM and BC_5_S_2_ plants enhanced their repellence properties against whiteflies. The use of jasmonates and other elicitors for plant defense induction has been described extensively in the literature (Dicke and Hilker, 2003). Application of the elicitor MeJA can establish direct and indirect plant resistance resulting in the reduction of herbivore development and survival (Thaler et al., 2002a; Rohwer and Erwin, 2008). When compared to BC_5_S_2_, however, MM leaves showed higher attractiveness to whiteflies even after MeJA treatments. This might be explained by the absence of effective constitutive and inducible resistance traits against whiteflies in MM, such as type-IV glandular trichomes and acylsucrose production, only present in BC_5_S_2_. In both genotypes, however, MeJA increased the phenolic content in leaf exudates. We hypothesized that this might be due to the increase in type-VI leaf glandular trichome densities in both MM and BC_5_S_2_ plants, since type-VI trichomes on adaxial leaf sides can be induced by MeJA (Boughton et al., 2005; Maes and Goossens, 2010) and produce and store different allelochemicals, i.e., terpenes, defensive proteins, and phenolic compounds (Wagner, 1991; Besser et al., 2009; McDowell et al., 2011). Interestingly, production of phenolics was higher in leaf exudates of BC_5_S_2_ plants. This might be in part associated with the induction of type-IV trichome densities and metabolism, as phenolic compounds were detected therein (**Figure 1D**). In tomato, leaf glandular trichomes and production of their associated allelochemicals play an important role in plant defenses (Kang et al., 2010a,b; McDowell et al., 2011; Glas et al., 2012) and, therefore, their induction might confer additional protection against herbivory. Deterrent properties of phenolics, however, have been reported for some herbivore species but not for whiteflies (Duffey and Isman, 1981; Mitchell et al., 1993; Kang et al., 2010a,b). Moreover, adult *B. tabaci* whiteflies locate preferentially on abaxial leaf surfaces (Freeman et al., 2001; Rodríguez-López et al., 2012). This indicates that higher production of phenolics by type-VI trichomes in adaxial leaf sides of MM plants might be ineffective to deter whitefly feeding. Nevertheless, JA-mediated induction of other defense and trichome-related compounds in MM, i.e., terpenes, might account for the enhanced repellence to whiteflies (Bleeker et al., 2009). For BC_5_S_2_, the role of type-IV trichomes-mediated production of phenolics in plant resistance against whiteflies is an aspect that would need further investigation.

Here, we also show that repeated MeJA applications resulted in reduced TYLCV-IL transmission by whiteflies in BC_5_S_2_ plants, but not in MM. Our previous studies reported on the generation of acylsucrose-producing backcross lines displaying high resistance to whiteflies and reduction of TYLCD incidence (Rodríguez-López et al., 2011). Acylsugar-producing type-IV glandular trichomes are known as effective barriers against aphids (Goffreda et al., 1989), spider mites (Fernández-Muñoz et al., 2000, 2003), leafminers (Hawthorne et al., 1992; Oliveira et al., 2012), and whiteflies (Firdaus et al., 2012; Leckie et al., 2012). Still, this trichome-based resistance is compromised in young developmental stages of our BC lines due to lower type-IV trichome densities and acylsucroses production (Rodríguez-López et al., 2011). Reinforcement of these constitutive defenses by treating young BC_5_S_2_ plants with MeJA might explain the observed differences in TYLCV transmission by whiteflies described here, in agreement with previous reports (Sippell et al., 1987; Walker, 1988; Snyder et al., 1998; Muigai et al., 2002; Sadasivan and Thayumanavan, 2003; Oriani and Vendramim, 2010). This increase in acylsucrose secretions is probably associated with the induction of type-IV glandular trichome density that is only present in BC_5_S_2_. Hence, though Maes and Goossens (2010) demonstrated that MeJA can induce higher densities of type-I trichomes in tomato, and this trichome-type is also reported to produce acylsugars (Schilmiller et al., 2010), leaf type-I trichomes were only and scarcely present in the main nerves of BC_5_S_2_ leaves (Supplementary Figure S2). Furthermore, acylsucroses were not detected in MM in our analysis. This confirms the idea that acylsugar levels produced in pest-susceptible tomato cultivars by type-I trichomes are insignificant when compared to those found in pest-resistant wild species (Alba et al., 2009; McDowell et al., 2011), and that, even after trichome induction, they are not really effective for pest control purposes.

Overall, our results strongly support that induced type-IV trichomes, as well as their secondary defense metabolism after MeJA treatments, accounted for the increased JA-induced resistance to whiteflies and reduced TYLCD transmission in BC_5_S_2_ plants. BC_5_S_2_ and MM are near-isogenic lines. Generation of BC_5_S_2_ was performed by recurrent backcrosses to MM and subsequent phenotypic selection of resistant plants based on type-IV glandular trichomes density and secretion of acylsucroses. This breeding process could result in the selection of genomic regions coding for trichome-associated traits from the wild donor while enriching the genetic background in genes from the susceptible parent MM. Differences in JA-induced defenses between BC_5_S_2_ and MM would be expected, therefore, to be largely centered around the activation of genes related to type-IV trichomes production and acylsucrose content in leaf exudates in the breeding line, absent in MM. Moreover, while type-IV trichome densities were approximately 1.9-fold induced after MeJA application in BC_5_S_2_ plants, acylsucrose content in the leaf exudates was increased 10 times (**Figures 1A,B**), suggesting a higher production of these compounds per trichome. To test this hypothesis we analyzed the transcriptomic responses of MM and BC_5_S_2_ plants to multiple MeJA applications at 21 dai. Comparative transcriptome analysis of these two near-isogenic lines revealed that overall the trichome-associated defenses were activated more strongly in the acylsugar producing line BC_5_S_2_ than in MM. This is not surprising since production and secretion of acylsugars is a complex phenotypic trait controlled by multiple genetic factors (Lawson et al., 1997). Several metabolic pathways are involved in their biosynthesis and, in agreement with this, numerous *quantitative trait loci* (QTL) located on different chromosomes have been associated with changes in acylsugar production and type-IV trichome density. Thus, distinct genetic configurations of these QTLs have been associated to changes in the sugar moiety, fatty acid esters composition, and total acylsugar production in wild tomato species (Mutschler et al., 1996; Blauth et al., 1998; Leckie et al., 2012, 2013).

Acylsugar biosynthesis results from a combined synthesis of branched chain amino acids (Leu, Val, and Ile), precursors for keto-acids biosynthesis, followed by their conversion into branched-chain acyl-CoAs by BCKD complex. These acyl-CoAs can act as substrates for the fatty acid synthase complex (FAS), reported as the major pathway responsible for providing medium-branched acylCoAs to acylsugar production and wax alkane biosynthesis (Slocombe et al., 2008). In addition, components of α-keto elongation (αKAE), associated with leucine precursor biosynthesis play an important role, as they provide precursors for keto-acids biosynthesis (Kroumova and Wagner, 2003; Ning et al., 2015). In line with this, genes involved in lipid and amino acid metabolism were among the enriched categories up-regulated in MeJA-treated BC_5_S_2_. Next, transfer of acyl chains to sucrose has been proposed to occur mainly via an Acetyl-coA dependent pathway (Schilmiller et al., 2012, Schilmiller et al., 2015). Accordingly, in the microarray analysis, two type-IV/I trichome-specific BAHD acyltransferases (*Sl-ASAT2* and *Sl-ASAT3*) (Schilmiller et al., 2015; Fan et al., 2016) were induced in MeJA-treated BC_5_S_2_ plants and showed higher expression levels when compared to MeJA-treated MM plants. Our time-course experiments confirmed the induction of some of the above-mentioned acylsugar-related biosynthetic genes after sequential MeJA applications. We showed that the component of the BCKD complex, *BCKD-E_2_* gene, was highly up-regulated after each hormone treatment when compared to MM (**Figure 5A**). Similarly, other key components of this metabolic pathway, *BCKD-E_3_* and *FAE-3*, were induced, emphasizing the successful transfer of constitutive, but also inducible, *S. pimpinellifolium* resistant-associated genes into the susceptible *S. lycopersicum* cultivar. By contrast, *FAE-3*, involved in fatty acid elongation, and *TD* and *IPSM-1* genes, responsible for the biosynthesis of keto-acid precursors, did not show higher expression levels at 15 dai. Moreover, in the case of *TD* and *IPSM-1* genes, they were down-regulated after hormone applications at 7 and 15 dai, respectively. Differences in expression of *FAE-3, TD* and *IPSM-1* genes between the microarray and RT-qPCR analyses might be explained by the different time point at which plants were sampled, 21 and 15 dai, respectively. Overall, these results also suggest that in BC_5_S_2,_ MeJA positively induced the acylsugar biosynthesis pathway mainly by prioritizing the production of AcylCoAs through the activation of BCKD complex. Interestingly, *BCKD-E_2_* showed higher and faster inductions after pre-treatment of BC_5_S_2_ plants with MeJA. An increased density of type-IV trichomes might account for this stronger expression of *BCKD-E_2_*. Nevertheless, significant higher trichome densities were only detected at 21 dai in preliminary experiments, suggesting that the positive regulation of the acylsugar biosynthesis machinery might occur within pre-existing type-IV trichomes at 15 dai. We hypothesize that higher expression levels of *BCKD-E_2_* after previous MeJA treatments might be explained by a priming effect. Baldwin and Schmelz (1996) reported that previous treatment of *Nicotiana sylvestris* plants with MeJA gave rise to more rapid nicotine accumulation after induction compared (Schilmiller et al., 2015) to untreated plants. This form of sensitization is referred to as ‘priming’ and can be established by a treatment with defense elicitors, e.g., chemicals, but also previous contact with necrotizing pathogens, mycorrizhal fungi and herbivores, that enables plants to display a more robust and accelerated induced defense responses upon subsequent herbivore or pathogen attack (Engelberth et al., 2004; Conrath et al., 2006; Frost et al., 2008). Whether enhanced acylsucrose secretions in BC_5_S_2_ are influenced by JA-mediated priming will need further analysis. The relevant role of acylsucroses in plant defense, and the fact that a strong response in their biosynthesis machinery upon previous JA-mediated activation might occur, provides new opportunities for utilizing this trait in plant protection. Additionally, among the differences between MM and BC_5_S_2_ we observed that the photosynthetic apparatus gene set was down-regulated stronger in BC_5_S_2_. In tomato, MeJA has been reported to reduce the photosynthetic capacity of treated leaves and increase N:C ratio, a signal that can stimulate production of defensive compounds involved in direct and indirect plant defenses (Gómez et al., 2010). This response might be related to the stronger induction of defense secondary metabolites, such as acylsucroses, in MeJA-treated BC_5_S_2_ plants.

Finally, our study also showed that MeJA-induced BC_5_S_2_ plants activated JA-mediated responses and induced even higher production of acylsucroses at 14 days after whitefly infestation (**Figure 6A**), providing therefore a durable protection. It is known that the whitefly *B. tabaci* can alter plant hormone-mediated responses in infested tomato plants by promoting induction of SA-mediated defenses while suppressing JA defense responses (Zarate et al., 2007; Estrada-Hernández et al., 2009; Puthoff et al., 2010). Whiteflies are phloem feeding insects alleged to be experts in avoiding and manipulating JA-mediated plant defense responses. Adult whiteflies avoid induction causing little damage in plant tissues, and nymphs manipulate induction by activating (harmless) SA-responses, which antagonize the (harmful) JA-responses (Zarate et al., 2007; Walling, 2008; Zhang et al., 2009). Then, it would be possible that previous JA-induction in BC_5_S_2_ plants might have altered the effectiveness of adult whiteflies to dodge and avoid their detection. Prior to feeding, whiteflies use their stylets to make shallow probes on the leaf surface of plant hosts, and if they detect physical and/or chemical defense barriers, not appropriate for colonization, they leave and search for another host (Walling, 2008; Bleeker et al., 2012). Whiteflies might have induced JA-mediated plant responses during these shallow probes in MeJA-treated BC_5_S_2_ plants. Yet, it is also unknown whether a more hairy leaf surface will sense a herbivore more easily via trichomes being ruptured (Peiffer et al., 2009). In addition, the effectiveness of the JA-mediated induction of acylsucrose-producing trichomes against other pests/pathogens that do not avoid activation of the JA signaling pathway (Thaler et al., 2002b; Zhao et al., 2003), but whose colonization might be affected by the trichome defenses, not present in cultivated tomatoes, would need further research.

## Author Contributions

RE-B, RF-M, EM, JA, and MK designed the research. RE-B performed the hormone, insect and virus experiments, chemical analyses and RT-qPCRs. Data analysis and interpretation was performed by RE-B, RF-M, EM, JA, MK, CP, and AG. Microarray experiment was performed by CP and its analysis and interpretation was made by CP, RE-B, AG, and MK. RE-B drafted the manuscript with critical review by all the authors.

## Conflict of Interest Statement

The authors declare that the research was conducted in the absence of any commercial or financial relationships that could be construed as a potential conflict of interest.

## Footnotes

1 www.ebi.ac.uk/arrayexpress
