# Supplementary material for: A Jasmonate-Inducible Defense Trait Transferred from Wild into Cultivated Tomato Establishes Increased Whitefly Resistance and Reduced Viral Disease Incidence
Source: Front Plant Sci. 2016 Nov 22;7:1732. doi: 10.3389/fpls.2016.01732 (PMC5118631; doi:10.3389/fpls.2016.01732)
Supplement: Supplementary file 1 [file Presentation_1.ZIP › Supplementary Figure 1.doc]

Supplementary Material

# A jasmonate-inducible defense trait transferred from wild into cultivated tomato establishes increased whitefly resistance and reduced viral disease incidence

Rocío Escobar-Bravo, Juan M. Alba, Clara Pons, Antonio Granell, Merijn R. Kant, Enrique Moriones and Rafael Fernández-Muñoz*

*** Correspondence:** Rafael Fernández-Muñoz: [rafael.fernandez@ihsm.uma-csic.es](mailto:rafael.fernandez@ihsm.uma-csic.es)

**Supplementary Figure 1**

**Supplementary Figure 1.** Scheme of the breeding program for pest resistance derived from *Solanum pimpinellifolium* accession TO-937 at IHSM La Mayora UMA-CSIC. Development of advanced backcross lines derived from the initial cross between the susceptible tomato *S. lycopersicum* `Moneymaker´ and TO-937 in which type IV glandular trichomes and acylsucrose secretion are introduced into the `Moneymaker´ genetic background to give the BC_5_S_2_ line used in this study.
