# Supplementary material for: A Jasmonate-Inducible Defense Trait Transferred from Wild into Cultivated Tomato Establishes Increased Whitefly Resistance and Reduced Viral Disease Incidence
Source: Front Plant Sci. 2016 Nov 22;7:1732. doi: 10.3389/fpls.2016.01732 (PMC5118631; doi:10.3389/fpls.2016.01732)
Supplement: Supplementary file 1 [file Presentation_1.ZIP › Supplementary Figure 2.doc]

Supplementary Material

# A jasmonate-inducible defense trait transferred from wild into cultivated tomato establishes increased whitefly resistance and reduced viral disease incidence

Rocío Escobar-Bravo, Juan M. Alba, Clara Pons, Antonio Granell, Merijn R. Kant, Enrique Moriones and Rafael Fernández-Muñoz*

*** Correspondence:** Rafael Fernández-Muñoz: [rafael.fernandez@ihsm.uma-csic.es](mailto:rafael.fernandez@ihsm.uma-csic.es)

**Supplementary Figure 2**

**Supplementary Figure 2.** Scanning electron micrographs of abaxial leaf surfaces from the acylsucrose-producer breeding line BC_5_S_2_ (A-B), and the pest and pathogen susceptible tomato cultivar `Moneymaker´ (MM) (C-D). Abaxial leaf surfaces of BC_5_S_2_ plants are covered by non-glandular (V) and glandular trichomes (I, IV, and VI), being type IV glandular trichomes (yellow triangles) chiefly responsible of acylsucrose secretions in this breeding line. Type I glandular trichomes (white triangles) were only present in the main vein of BC_5_S_2_ leaves. MM abaxial leaf surface a high-density of type V non-glandular trichomes is observed (C), with less proportion of type I and (D) VI glandular trichomes. Type IV glandular trichomes (E) are absent in MM. White horizontal bars represent 100 μm in panel (A), (C), and (D), and 200 μm in panel (B) and (E).
