# Supplementary material for: A Jasmonate-Inducible Defense Trait Transferred from Wild into Cultivated Tomato Establishes Increased Whitefly Resistance and Reduced Viral Disease Incidence
Source: Front Plant Sci. 2016 Nov 22;7:1732. doi: 10.3389/fpls.2016.01732 (PMC5118631; doi:10.3389/fpls.2016.01732)
Supplement: Supplementary file 1 [file Presentation_1.ZIP › Supplementary Figure 3.doc]

Supplementary Material

# A jasmonate-inducible defense trait transferred from wild into cultivated tomato establishes increased whitefly resistance and reduced viral disease incidence

Rocío Escobar-Bravo, Juan M. Alba, Clara Pons, Antonio Granell, Merijn R. Kant, Enrique Moriones and Rafael Fernández-Muñoz*

*** Correspondence:** Rafael Fernández-Muñoz: [rafael.fernandez@ihsm.uma-csic.es](mailto:rafael.fernandez@ihsm.uma-csic.es)

**Supplementary Figure 3**

**control**

**MeJA**

**Supplementary Figure 3.** Validation of the microarray experiment. Expression analysis by real time RT-PCR of *Sesquiterpene synthase* (*TPS)*, *Cytochrome P450* (*CYP450*)*,* *Homeobox leucine zipper protei*n (HLZP) and the *ABC-transporter G family member-11* (*ABCG11*) genes in mock-treated and methyl jasmonate (MeJA) -treated MM and BC_5_S_2_ plants at 21 d after an initial hormone treatment. Plants received three successive MeJA applications, at days 0, 7, and 14. The mean fold induction (+ SEM, *n* = 3) is shown. RNA samples were the same used for Microarray analysis. Three biological replicates and two technical replicates were analyzed and normalized to *Actin* gene*.* To normalize the data and stabilize the variance normalized expression data were Log transformed prior statistical analysis. Significant differences between treatments were determined by *t-*test. Asterisks denote significant differences between treatments at *P <* 0.05.
