# Supplementary material for: A Jasmonate-Inducible Defense Trait Transferred from Wild into Cultivated Tomato Establishes Increased Whitefly Resistance and Reduced Viral Disease Incidence
Source: Front Plant Sci. 2016 Nov 22;7:1732. doi: 10.3389/fpls.2016.01732 (PMC5118631; doi:10.3389/fpls.2016.01732)
Supplement: Supplementary file 1 [file Presentation_1.ZIP › Supplementary Method 1.doc]

Supplementary Material

# A jasmonate-inducible defense trait transferred from wild into cultivated tomato establishes increased whitefly resistance and reduced viral disease incidence

Rocío Escobar-Bravo, Juan M. Alba, Clara Pons, Antonio Granell, Merijn R. Kant, Enrique Moriones and Rafael Fernández-Muñoz*

*** Correspondence:** Rafael Fernández-Muñoz: [rafael.fernandez@ihsm.uma-csic.es](mailto:rafael.fernandez@ihsm.uma-csic.es)

**Supplementary Method 1**

**Supplementary Method 1**

**Total phenolic compounds determination and staining**

Total leaf surface phenolic compounds were extracted using a modified method described in Antonious et al. (2003). In short, two leaflets from the third youngest leaf were excised, rinsed in 5 ml of 80% ethanol solution, and shaken for 1 h using an electric shaker. Leaflets were removed and area was measured with a Delta-T Area Meter MK2 (Cambridge, UK). Determination of phenolic compounds was performed using the Folin-Ciocalteu colorimetric method (Hagerman et al., 1997). Chlorogenic acid (Sigma-Aldrich) was used to obtain a standard calibration curve in the range of 1.75 to 14 μg ml-1 in 80 % ethanol solution and determine phenols as chlorogenic acid equivalent. MeJA-treated and control plants were sampled for detection of flavonoid compounds in the abaxial leaf surfaces with saturated (0.25% w/v) diphenylboric acid-2- aminoethyl ester (DPBA) (Sigma-Aldrich) with 0.02% Triton X-100. Flavonoids can be visualized locally *in planta* due to their natural fluorescence properties, but addition of DPBA, which complexes with flavonoids, enhances their fluorescence properties. Moreover, the fluorescence color of the DPBA-stained phenylpropanoids provides additional information about their basic chemical structure, having a characteristic green fluorescence when kaempferol derivatives are detected and gold/orange fluorescence in the case of detection of quercetin derivatives (Buer et al., 2007, 2010). A light/epifluorescence microscope (Optiphot, Nikon) equipped with a mercury lamp (Nikon, HB-10101AF) as an UV light source and UV excitation filter (340-380 nm) was used to visualize DPBA-flavonoid fluorescence. Images were captured using a camera Nikon AFX adapted to the microscope.
