# Supplementary material for: A Jasmonate-Inducible Defense Trait Transferred from Wild into Cultivated Tomato Establishes Increased Whitefly Resistance and Reduced Viral Disease Incidence
Source: Front Plant Sci. 2016 Nov 22;7:1732. doi: 10.3389/fpls.2016.01732 (PMC5118631; doi:10.3389/fpls.2016.01732)
Supplement: Supplementary file 1 [file Presentation_1.ZIP › Supplementary Method 2.doc]

Supplementary Material

# A jasmonate-inducible defense trait transferred from wild into cultivated tomato establishes increased whitefly resistance and reduced viral disease incidence

Rocío Escobar-Bravo, Juan M. Alba, Clara Pons, Antonio Granell, Merijn R. Kant, Enrique Moriones and Rafael Fernández-Muñoz*

*** Correspondence:** Rafael Fernández-Muñoz: [rafael.fernandez@ihsm.uma-csic.es](mailto:rafael.fernandez@ihsm.uma-csic.es)

**Supplementary Method 2**

**Supplementary Method 2**

**Microarray analysis**

Data were pre-processed and analyzed using Partek Genomic Suite software v6.6 (Partek Inc., St. Louis, MO) with the probes matching only once with the iTAG annotation v2.3. The configuration consisted of a pre-background adjustment for GC content, Robust Multi-array Analysis (Irizarry 2003) for background correction, quantile normalization and probe set summarization using median polishing. All signals were log2 transformed. Library files were eutom3gene_v2_ucprobes.cdf and the annotation file version was eutom3 annotaions17-02-12.txt which represents 30.000 genes of tomato genome*.*

To identify genes up or down regulated by MeJA in BC_5_S_2_ and MM samples probe set information is summarized into gene level information. A 3-way ANOVA model by using Method of Moments to analyze the effects of genotype, chemical treatment, replicate and interaction between type and sample type. The ANOVA model was:

Y_ijkl_ = μ + genotype_i_ + Chemical treatment_j_ + sample type (genotype * Chemical treatment)_ijk_+ ε_ijkl_

Where Y_ijkl_ represents the lth observation on the ith genotype j^th^ Chemical treatment k^th^ sample type. The mean μ is the common effect for the whole experiment. ε_ijkl_ represents the random error present in the l^th^ observation on the i^th^ genotype j^th^ Chemical treatment k^th^ sample type. The errors εijkl are assumed to be normally and independently distributed with mean 0 and standard deviation δ for all measurements.

To determine specific group differences in case of significant main effects (or interaction), the ANOVA was followed by Fisher’s LSD post hoc contrast to generate p-values and fold changes for comparisons. Gene lists of pair-wise contrasts were divided into up- and down-regulated genes (compared with its control). Subsequently, genes with a p-value less than 0.05, and an absolute fold change larger than 1 (2 fold in actual value), were defined as differentially expressed.

Genes were selected to explain the "extra" induced resistance in BC_5_S_2_ after MeJA application as compared the MM genotype because they satisfy two conditions: 1) they should be found significantly affected by the treatment (ANOVA-treatment p-value <0.05) and differentially expressed in BC5, in MM or both after MeJA treatment (FC>1 and FDR<0.05) and 2) they should show expression differences when BC_5_S_2_-MeJA and MM-MeJA are compared larger than 1.5 fold.

The identified differentially expressed genes into clusters were used to calculate MapMan term enrichment scores against molecular function categories by applying Fisher Exact tests using a local, customized version of the 'catscore.pl' Perl script described in Cheung et al. (2003) was used. Only MapMan terms with a p <0.05, and more than three regulated genes for the Mapman-term were defined as over-represented.
