# Supplementary material for: A Jasmonate-Inducible Defense Trait Transferred from Wild into Cultivated Tomato Establishes Increased Whitefly Resistance and Reduced Viral Disease Incidence
Source: Front Plant Sci. 2016 Nov 22;7:1732. doi: 10.3389/fpls.2016.01732 (PMC5118631; doi:10.3389/fpls.2016.01732)
Supplement: Supplementary file 1 [file Presentation_1.ZIP › Supplementary Table 1.doc]

Supplementary Material

# A jasmonate-inducible defense trait transferred from wild into cultivated tomato establishes increased whitefly resistance and reduced viral disease incidence

Rocío Escobar-Bravo, Juan M. Alba, Clara Pons, Antonio Granell, Merijn R. Kant, Enrique Moriones and Rafael Fernández-Muñoz*

*** Correspondence:** Rafael Fernández-Muñoz: [rafael.fernandez@ihsm.uma-csic.es](mailto:rafael.fernandez@ihsm.uma-csic.es)

**Supplementary Table 1**

**Supplementary Table 1. Primers used for real time RT-PCR assays of relative gene expression.**

| **Primer name** | **Gene name/accession no.** | **Primer sequence (5´-3´, forward, reverse)** |
| --- | --- | --- |
| *TD* | *Threonine dehydratase/deaminase*  (Solyc10g083760) | CCGCCGTGAAATCATTGAAT GCTAATAGGAACAATGACGGAACTC |
| *FAE* | *Fatty acid elongase 3-ketoacyl-CoA synthase*  (Solyc10g009240) | TCGTTCAGGTTTAGGTGACG  CGTGCCTCTTTCATGTTGAG |
| *IPMS-1* | *2-Isopropylmalate synthase 1*  (Solyc06g053400) | ATGACCTGTTCTGGCGATTC  TGAGGCTGGAAAACCTCATC |
| *BCKD-E_3_* | *Dihydrolipoyl dehydrogenase*  (Solyc12g099100) | TTGCAGTACGGAGCATCAAG  CATGGCTGCTTCTTTGAGTG |
| *BCKD-E_2_* | *Dihydrolipoyllysine-residue acetyltransferase or Branched-chain α-keto acid dehydratase* (E2 subunit) (Solyc01g066520) | TGTCGAAGAGATAAATTGTGATGC  TGAGGACGGGAAGGAAAGTG |
| *WIPI-II* | *Wound inducible proteinase inhibitor II/* (Solyc01g095200) | GACAAGGTACTAGTAATCAATTATCC GGGCATATCCCGAACCAAGA |
| *Actin* | *Actin /*  (Solyc03g078400) | TTAGCACCTTCCAGCAGATGT  AACAGACAGGACACTCGCACT |
