# Supplementary material for: A Jasmonate-Inducible Defense Trait Transferred from Wild into Cultivated Tomato Establishes Increased Whitefly Resistance and Reduced Viral Disease Incidence
Source: Front Plant Sci. 2016 Nov 22;7:1732. doi: 10.3389/fpls.2016.01732 (PMC5118631; doi:10.3389/fpls.2016.01732)
Supplement: Supplementary file 1 [file Presentation_1.ZIP › Supplementary Table 2.doc]

Supplementary Material

# A jasmonate-inducible defense trait transferred from wild into cultivated tomato establishes increased whitefly resistance and reduced viral disease incidence

Rocío Escobar-Bravo, Juan M. Alba, Clara Pons, Antonio Granell, Merijn R. Kant, Enrique Moriones and Rafael Fernández-Muñoz*

*** Correspondence:** Rafael Fernández-Muñoz: [rafael.fernandez@ihsm.uma-csic.es](mailto:rafael.fernandez@ihsm.uma-csic.es)

**Supplementary Table 2**

***Supplementary Table 2.*** *The accession numbers and primer sequences for the validation genes from microarray analysis.*

| **Primer name** | **Gene name/ accession no.** | **Primer sequence (5´- 3´, forward, reverse)** | **Gene expression** |
| --- | --- | --- | --- |
| *TPS* | *Sesquiterpene synthase* Solyc07g052150 | CGTCGATGTGCCAATCATCA CCATTCATTGGCTCCCAAGAGA | Up-regulated only in MeJA-treated BC_5_S_2_ |
| *CYP450* | *Cytochrome P450*  Solyc10g078220 | ATGTCCAGGAATGAACCTTGC  GTGCCAGGACTCTCCATCAT | Up-regulated only in MeJA-treated BC_5_S_2_ |
| *HLZP* | *Homeobox leucine zipper protei*n  Solyc01g096320 | AACAACGCGAGGAGGTTCAG  TCCGAGTTCTCTGGCTAGTTG | Common down-regulated in MeJA-treated BC_5_S_2_ and MM |
| *ABCG11* | *ABC-transporter G family member 11*  Solyc01g105450 | GCCAGCTTCACAACTCAAAG TAAGCGCAGCCAGTAGTATC | Common down-regulated in MeJA-treated BC_5_S_2_ and MM |
